# Mitophagy in neuronal health and disease: from mechanisms to neurodegeneration

**DOI:** 10.1172/JCI199847

**Published:** 2026-09-01

**Authors:** Bishal Basak, Julia F. Riley, Neha M. Nataraj, Erika L.F. Holzbaur

**Affiliations:** 1 Department of Physiology, University of Pennsylvania Perelman School of Medicine, Philadelphia, Pennsylvania, USA.; 2 Aligning Science Across Parkinson’s (ASAP) Collaborative Research Network, Chevy Chase, Maryland, USA.

## Abstract

Regulation of mitochondrial health is critical for maintaining cellular homeostasis in the nervous system. Damaged mitochondria can have detrimental effects on neuronal health and are thought to be key contributors to the progression of neurodegenerative disorders including Parkinson’s disease and amyotrophic lateral sclerosis. To mitigate this damage, multiple quality control mechanisms have evolved to eliminate aged or damaged mitochondria. One such quality control process is autophagy, a process that involves turnover of mitochondria at presynaptic sites and the axon terminal under basal conditions. This highly conserved mechanism sequesters mitochondria from the cytosol within autophagosomes followed by degradation upon fusion with a lysosome. Acute mitochondrial damage activates a selective form of autophagy called mitophagy that involves receptor-mediated engulfment and degradation of the damaged organelle. Multiple mechanisms have been shown to drive efficient mitophagy in neurons and glia, including PTEN induced kinase 1 (PINK1)/Parkin–dependent mitophagy and receptor-mediated mitophagy. Genetic, pathological, and experimental evidence all implicate defects in the removal of damaged mitochondria in the onset or progression of neurodegenerative disease. Both the initiation of PINK1/Parkin–dependent mitophagy and deficits in the removal of damaged mitochondria are linked to activation of neuroinflammatory pathways, including NF-κB and cyclic GMP-AMP synthase–stimulator of interferon genes (cGAS-STING) signaling. In this Review, we discuss the molecular pathways governing mitophagy in neurons and glial cells and how deficits in these pathways may lead to neurodegeneration. We also highlight emerging therapeutic strategies aimed at restoring mitophagy to preserve neuronal homeostasis and function.

## Introduction

Mitochondria are central regulators of neuronal homeostasis, playing critical roles in cellular signaling, metabolic pathways, ATP production, synaptic transmission, and calcium buffering (1). Neurons are particularly reliant on mitochondrial function because of high bioenergetic demands. To facilitate this high demand, neurons are densely packed with mitochondria; for instance, a single neuron in the human substantia nigra is estimated to contain approximately 2 million mitochondria (2). The high metabolic flux typical of neuronal mitochondria makes these organelles particularly vulnerable to damage, especially oxidative stress. Thus, neurons rely on quality control mechanisms that facilitate the clearance of dysfunctional mitochondria to mitigate mitochondrial damage–induced neurotoxicity.

Mitochondrial turnover in the nervous system occurs via multiple pathways (3, 4), including basal turnover of mitochondria in the axon via autophagy, stress-induced turnover of damaged mitochondria by mitophagy, and piecemeal degradation of mitochondria. Genetic and pathological evidence link deficits in mitophagy to neurodegeneration, observations that motivated groundbreaking research into the molecular mechanisms driving mitophagy, including the discovery of PTEN induced kinase 1 (PINK1)/Parkin–dependent mitochondrial turnover (5), and work focusing on mitophagy in neurons (4). More recently, attention has turned to mitochondrial turnover in glia as an important contributing factor to neurodegenerative disease pathology. Going forward, a more holistic view of mitochondrial turnover is likely to be required, as mitochondrial quality control may not be cell autonomous in neurons or glia. For example, recent studies demonstrate that neuronal mitochondria may be off-loaded to other cell types for degradation (6, 7). There is also increasing interest in defining pathways by which mitochondrial damage or deficits in turnover trigger neuroinflammatory cascades (8, 9). Here, we discuss mitophagy in neurons and glia; review evidence linking deficits in mitochondrial turnover to neurodegeneration in Parkinson’s disease (PD), amyotrophic lateral sclerosis (ALS), and Alzheimer’s disease (AD); and highlight progress in the development of therapeutic strategies to mitigate deficits in mitophagy in the nervous system.

## Basal mitochondrial turnover in neurons

**Figure 1:**
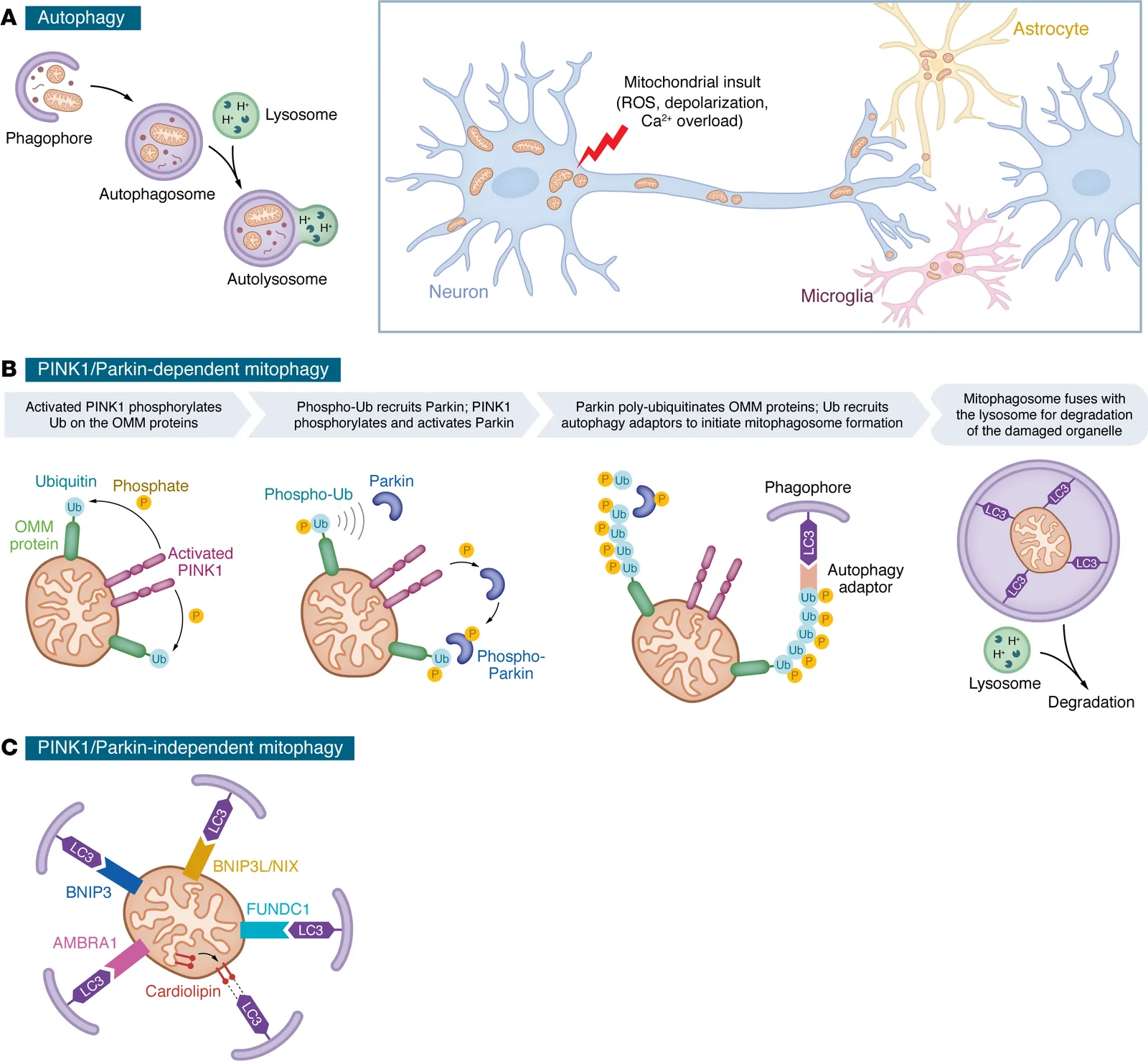
Mitochondrial quality control in neurons and glia. Schematic representation of a neuron, astrocyte, and microglia highlighting key mitochondrial quality control pathways. (**A**) Basal mitochondrial turnover via nonselective autophagy. (**B**) PINK1/Parkin–mediated mitophagy. (**C**) Receptor-mediated mitophagy that operates independently of PINK1/Parkin.

Under basal conditions, mitochondria in axons undergo turnover by constitutive autophagy in a tightly regulated and evolutionarily conserved pathway (10). Robust basal turnover of mitochondria has been observed in neurons in vitro and in vivo (11, 12). Under basal, unstressed conditions, neuronal mitochondrial turnover is initiated at presynaptic sites and the axon terminal, where mitochondrial fragments approximately 1 μm in diameter are engulfed by a double-membrane phagophore (Figure 1). Once cargoes are engulfed, autophagosomes are rapidly trafficked back to the soma for degradation. This transport is driven by cytoplasmic dynein and is activated by adaptors including JIP3/4, Huntingtin (HTT), and HAP1 (13, 14). Pathogenic mutations in *LRRK2* associated with PD or pathogenic expansion of glutamine repeats in HTT associated with Huntington’s disease inhibits the trafficking of axonal autophagosomes, impairing degradation of engulfed mitochondria (15, 16).

## PINK1/Parkin–dependent mitophagy

In contrast with the basal pathway described above, acutely damaged mitochondria are selectively sequestered into autophagosomes via receptor-mediated autophagy in a process termed mitophagy. PINK1/Parkin–mediated mitophagy is the best-characterized pathway for the targeted removal of damaged mitochondria (4) (Figure 1). PINK1 is a serine/threonine kinase constitutively imported into the inner mitochondrial membrane (IMM), where it is degraded by the protease presenilin-associated rhomboid-like protein (17). Upon mitochondrial insult, PINK1 import is arrested at the outer mitochondrial membrane (OMM), where it remains associated with the translocase of the outer mitochondrial membrane (TOM) complex (18). PINK1 then undergoes dimerization and autophosphorylation, leading to kinase activation. Activated PINK1 phosphorylates ubiquitin (Ub) bound to OMM proteins (19, 20). Phosphorylated Ub recruits the RING-between-RING E3 ubiquitin ligase Parkin, which is also phosphorylated by PINK1 (21). This activates Parkin’s E3 ligase activity, leading to rapid ubiquitination of OMM proteins including mitofusin-2 (MFN2), mitochondrial Rho GTPase 1 (MIRO1), MIRO2, TOM20, TOM70, CISD1, and VDAC (22).

Parkin-dependent Ub chains recruit Ub-binding autophagy adaptors including optineurin (OPTN), sequestosome 1 (SQSTM1; also called p62), calcium-binding and coiled-coil domain-containing protein 2 (also called nuclear dot protein 52 kDa or NDP52), Tax1 binding protein 1, and neighbor of BRCA1 gene 1 protein (23–25). These adaptors also possess LC3 interacting region (LIR) motifs that facilitate interactions with LC3-II, a lipidated protein associated with the membrane of the forming autophagosome (4). Among the five autophagy receptors, OPTN is highly expressed in the brain and is the predominant receptor for mitophagy in neurons (24). OPTN recruitment is enhanced via phosphorylation by the kinase TANK binding kinase 1 (TBK1) (26, 27). Both OPTN and NDP52 form sheet-like condensates on ubiquitinated mitochondria, facilitating organelle engulfment by LC3-II–positive autophagosomes (28) to form a mitophagosome. This process is sufficient to sequester damaged mitochondria from the cytosol; degradation of the internalized mitochondrial fragments requires subsequent fusion with a lysosome. This fusion step and the resulting breakdown of engulfed mitochondria are tightly regulated in neurons by factors including the Rab7-binding protein Rubicon (29–31).

Several upstream factors also govern the initiation and tight regulation of mitophagy. PINK1 promotes dynamin-related protein 1 (DRP1) recruitment to the mitochondria to drive mitochondrial fission and promote the physical segregation of the damaged organelle from the healthy network (32). Parkin-dependent ubiquitination of the mitochondrial fusion protein MFN2 targets it for valosin-containing protein (VCP)/p97–dependent extraction and proteasomal degradation, preventing the damaged organelle from re-fusing with the cellular network (33). PINK1 and Parkin can also induce mitochondrial biogenesis by promoting degradation of PARIS (ZNF746), a transcriptional repressor that otherwise blocks expression of PGC-1α, a master regulator of mitochondrial biogenesis genes (34). These findings indicate that mitophagy is not an isolated cellular pathway but is tightly integrated with broader mitochondrial dynamics, such as biogenesis, fission, and fusion. The importance of mitophagy to neuronal homeostasis is further reflected by the identification of mutations in genes of this pathway in patients with familial PD (*PINK1*, *PRKN*) and ALS (*OPTN*, *TBK1*, *SQSTM1*).

## PINK1/Parkin–independent mitophagy

Mitophagy pathways functioning independently of PINK1/Parkin have also been identified. These pathways are often mediated by receptors at the OMM that promote autophagosomal engulfment of mitochondria, including FUNDC1 (FUN14 domain-containing protein 1), AMBRA1 (autophagy and Beclin 1 regulator 1), BNIP3L (BCL2 interacting protein 3 like)/NIX, and BNIP3. The mitochondrial lipid cardiolipin has also been implicated in the initiation of PINK1/Parkin–independent mitophagy (35) (Figure 1).

FUNDC1 is an OMM protein harboring an LIR motif shown to initiate autophagosome formation (36). In vitro studies suggest a role for FUNDC1 in mitophagy induced under hypoxic conditions (36, 37). In vivo, FUNDC1 enhances mitophagy and reduces neuronal apoptosis in injured spinal cord tissues (38). FUNDC1 levels are reported to be higher in autophagosomes isolated from *Pink1*^–/–^ mice (39). In PINK1-mutant flies, expression of human FUNDC1 rescued mitochondrial defects and restored morphological and locomotory defects (40).

AMBRA1 was first identified as critical for the development of the nervous system in mouse embryos (41). AMBRA1 is abundant in adult brain, including in midbrain dopaminergic neurons that are affected in PD (42). Reduced levels of AMBRA1 impair neural tube development and autophagy, leading to increased apoptotic cell death, metabolic dysfunction, and protein aggregation, while triggering retinal degeneration (41, 43). Upon mitochondrial insult, AMBRA1 localizes around juxtanuclear clusters of the damaged organelles, where it stimulates phagophore formation by activating class III PI3K (42). AMBRA1 can interact with Parkin, but its role in PINK1/Parkin–mediated mitophagy is debated. While one study showed that AMBRA1-mediated activation of class III PI3K is Parkin dependent (42), others have reported that AMBRA1 can directly bind to LC3 (44) and promote mitophagy independent of Parkin (44, 45).

BNIP3L/NIX and its homolog BNIP3 are OMM proteins identified as interactors of antiapoptotic proteins BCL2 and E1B 19 kDa (46, 47). BNIP3L harbors an LIR motif that facilitates sequestration of mitochondria by an autophagosome (48, 49). BNIP3L expression is induced by HIF-1α (50) under hypoxic conditions to upregulate mitophagy (51, 52). In the brain, BNIP3L-dependent mitophagy has been reported to reduce damage caused by ischemic stroke (52). However, BNIP3L was found to be dispensable for mitophagy under oxidative stress conditions (53). Like BNIP3L/NIX, BNIP3 functions as an autophagy receptor promoting mitophagy under hypoxic conditions (50). BNIP3 expression is enhanced in *Pink1*^–/–^ mice (39), indicating the possibility of a compensatory pathway in rodents.

Receptor-mediated mitophagy may function in concert with changes in mitochondrial lipids. Cardiolipin is a mitochondrially localized lipid demonstrated to regulate mitophagy. Under basal conditions, cardiolipin constitutes approximately 20% of the total lipid content in the IMM and about 3% of the OMM (54). Upon mitochondrial damage, cardiolipin is redistributed to the OMM, where it directly interacts with LC3 to stimulate mitophagosome formation (55). Cardiolipin redistribution may also facilitate receptor-driven mitophagy involving NIX, BNIP3, or FUNDC1 (56).

E3 ligases, such as mitochondrial ubiquitin ligase 1 (MUL1), can also initiate mitophagy. MUL1, localized to the OMM, promotes mitochondrial fission by SUMOylating the mitochondrial fission protein DRP1, facilitating its recruitment and activity (57). MUL1 can complement Parkin function by ubiquitinating the latter’s substrate, MFN2, to initiate mitophagy in the absence of Parkin (58). Consistent with this, depletion of MUL1 in *Parkin^–/–^* neurons or in *Drosophila* additively impairs mitochondrial health and induces neurodegeneration (58). Loss of MUL1 in neurons results in abnormal, hyperfused mitochondria with altered dynamics and function (58).

## Mitophagy in neurons

In vivo studies demonstrate robust clearance of damaged mitochondria by autophagy in neurons (11, 59), mediated by PINK1/Parkin–dependent and independent pathways. In vitro, in neurons subjected to mild oxidative stress, mitophagy occurs primarily in the soma or presynaptic varicosities (29, 60). The initial steps in the pathway — activation of PINK1/Parkin, ubiquitination of OMM proteins, recruitment of OPTN, and assembly of an LC3-positive autophagosome — occur with kinetics similar to observations in non-neuronal cells expressing Parkin (27, 29). Surprisingly, however, maturation of the resulting mitophagosomes and degradation of engulfed mitochondria are much slower in neurons than other cell types and are rate-limiting in mitochondrial turnover (29). This delayed maturation is due, at least in part, to expression of Rubicon, which negatively regulates lysosome acidification and restricts mitophagosome-lysosome fusion (30). Consistent with this, depletion of Rubicon accelerates mitochondrial turnover in neurons (30).

Despite strong evidence that PINK1/Parkin–dependent mitophagy is induced in neurons in response to acute mitochondrial damage, the contribution of this pathway to the regulation of neuronal homeostasis differs across species. Whereas loss of *Pink1* or *Parkin* contributes to defects in mitophagy in *Drosophila*, triggering degeneration of dopaminergic neurons (61, 62), knockout of either *Pink1* or *Parkin* in mice has little effect on mitophagy under basal conditions and does not induce marked neuronal loss or a discernible phenotype (11, 63, 64). In aged mice, however, loss of PINK1 induces mitochondrial damage and reduced dopamine release (65, 66). Loss of PINK1 or Parkin in monkeys triggers degeneration of the dopaminergic neurons with age, consistent with age-dependent roles of this pathway in the maintenance of neuronal health (67, 68). In humans mutations in the gene encoding Pink1 and Parkin are causal for familial PD, as discussed in detail below.

## Mitophagy in glia

While mitophagy has long been appreciated as important for neuronal function, an in-depth focus on mitophagy in glial cells, such as microglia and astrocytes, is more recent. This emergent research marries a process that mediates cellular inflammation via clearance of damaged mitochondria with the cell types most heavily implicated in the neuroinflammatory component of neurodegenerative diseases (69, 70).

Microglia, often deemed the brain’s immune cells, have the capacity to clear protein aggregates via the energy-demanding process of phagocytosis (71). In microglia, autophagic clearance of mitochondria has been observed in vivo in the cortex and hippocampus (11). Enhancement of mitophagy through treatment with urolithin A (discussed below) was found to increase the capacity of these cells to phagocytose amyloid-β (Aβ) plaques in an AD model (72). Urolithin A treatment in a PD mouse model alleviated motor symptoms and neurodegeneration (6). This finding was attributable to microglial mitophagy, as microglia-specific depletion of a protein essential for canonical autophagy (ATG5) ameliorated the benefit of the treatment (73). Together, these studies suggest that promoting mitophagy in microglia aids their capacity to perform neuroprotective functions.

A growing number of studies indicate that persistent mitochondrial damage and/or defective mitophagy in microglia can actively promote pro-inflammatory signaling (74, 75). Loss of PINK1 was implicated in promoting microglial production of pro-inflammatory cytokines both at baseline (76) and in a mouse model of intracerebral hemorrhage (77). Enhanced TNF-α production was observed in microglia from an *App/Ps1* mouse model of AD, while cytokine production was decreased upon mitophagy activation in a PINK1-dependent manner (72). Thus, while promoting mitophagy in microglia appears to sustain their neuroprotective capacity, loss of mitophagy in these cells may actively promote neuroinflammatory signaling that exacerbates neurodegenerative phenotypes.

Astrocytes are best known as support cells that provide critical support to neurons throughout development and aging. In vitro studies on primary astrocytes indicate lysosomal clearance of damaged mitochondria via PINK1 activation (78, 79). In vivo, mitophagy has been observed in glial fibrillary acidic protein–positive (GFAP^+^) astrocytes in mouse brain (11). GFAP is a marker of pan-reactive astrogliosis, indicative of one or more environmental factors causing astrocytes to transiently adopt a transcriptional regimen different from baseline activity (80). Reactive astrocytes can participate in neuroinflammation and have been observed in neurodegenerative diseases, including PD, ALS, and AD (80–82).

In astrocytes, mitophagy is closely linked to glycolytic machinery; PINK1 forms a complex with the enzyme hexokinase 2 (HK2) to facilitate phospho-Ub formation at the OMM, and HK2 knockdown blunts astrocytic mitophagy (83). Cytokine treatment of astrocyte-neuron cocultures induced increased neurotoxicity in the absence of PINK1 and HK2, suggesting that mitophagy in astrocytes reduces cytokine-induced neurotoxicity (83). Beyond classic PINK1/Parkin–dependent mitophagy, the C-type lectin domain containing protein 16A (CLEC16A) has also been identified as a mediator of mitophagy in astrocytes (84). CLEC16A expression limited pro-inflammatory responses in astrocytes following cytokine treatment, while *Clec16a* deletion in a mouse model of multiple sclerosis resulted in higher expression of pro-inflammatory proteins, such as gasdermin D, in reactive astrocytes (84). Together, these findings suggest the importance of mitophagy in quelling neurotoxic astrocyte responses to microglial signaling.

While astrocytic mitophagy appears to be neuroprotective, other work indicates that astrocytes, much like microglia, can actively promote inflammation should they fail to efficiently clear damaged mitochondria. Mitochondrial damage in astrocytes is sufficient to activate NF-κB signaling (78). Astrocytes with impaired oxidative phosphorylation also have a decreased capacity for β-oxidation of fatty acids, leading to an accumulation of lipids that can drive astrocyte reactivity and become neurotoxic (85). These studies demonstrate that loss of mitochondrial function can modulate reactive astrogliosis and, correspondingly, astrocytic participation in neurodegenerative disease.

## Mitophagy and inflammation

Links between impaired mitophagy and activation of inflammatory signaling pathways have been well documented across in vitro studies, animal models, clinical studies, and postmortem analyses (8, 86–88). Inflammatory signaling programs can regulate mitophagy (86, 87, 89), and conversely, suppression of mitophagy can lead to accumulation of damaged mitochondria, inducing inflammation (8, 86–88) via release of mtDNA into the cytosol and subsequent cyclic GMP-AMP synthase–stimulator of interferon genes (cGAS-STING) activation (8, 90, 91), increased cellular ROS (86, 91, 92), and inflammasome activation (86–88). Evidence that impaired mitophagy induces pathological inflammation has been observed across multiple disease models, including sepsis, cardiac inflammation, acute kidney injury, and neurodegeneration (86, 91, 93, 94). Furthermore, treatments to induce mitophagy, including urolithin A, rapamycin, and hypoxia-preconditioned exosomes, have been shown to alleviate inflammation (94–96).

A more limited form of mitochondrial turnover occurs when select areas of damaged mitochondria bud off to form mitochondrially derived vesicles (MDVs) that are targeted for degradation. Evidence suggests that suppression of mitophagy combined with oxidative stress can lead to upregulation of MDV formation; these MDVs can be generated in a Parkin-dependent manner and are then trafficked to and degraded by lysosomes (97, 98). In the absence of PINK1/Parkin activity, lysosomal and proteasomal processing of MDVs can be followed by mitochondrial antigen presentation, which can elicit inflammation and autoimmune disease (99, 100). Alternatively, in the absence of Parkin, MDVs or mitochondria themselves can be targeted to extracellular vesicles for secretion (7, 101, 102). Secretion of mitochondria and mtDNA represents a potential source of damage-associated molecular patterns with the capacity to induce non–cell-autonomous inflammatory signaling cascades under disease conditions.

The OMM can serve as a scaffold for cell signaling (103), including for recruitment of the innate immune signaling adaptor MAVS, which acts downstream of RIG-I-like receptor (RLR) activation. RLRs are a family of cytosolic pattern recognition receptors critical for host defense in response to viral dsRNA. Once activated, MAVS recruits either the IKK complex to initiate NF-κB signaling or TBK1 to induce interferon regulatory factor 3 signaling (104, 105). MAVS can also be activated by ROS (106). Furthermore, mitophagy and mitophagy regulators have been reported to curtail MAVS-induced inflammation (107–109), adding to the many roles mitophagy has in suppressing inflammation.

The OMM can also serve as a platform for activation of NF-κB signaling across different cell types (9, 110, 111). PINK1 and Parkin are known to promote NF-κB signaling, with various mechanisms proposed (112–115). In response to mitochondrial damage, Parkin-dependent K63 ubiquitylation of the OMM was shown to recruit the NF-κB essential modulator NEMO, which has a Ub-binding domain strikingly similar to the Ub-binding motif in the mitophagy adaptor OPTN (9). NEMO recruitment leads to IKK assembly and induction of downstream NF-κB signaling (9). A similar damage-induced recruitment of NEMO to Ub chains on mitochondria formed during apoptosis can also lead to NF-κB activation (111). Importantly, mitochondrial damage to astrocytes activates NF-κB signaling in addition to other pro-inflammatory signaling cascades, potentially driving neurotoxicity (78).

Thus, multiple studies demonstrate that damaged mitochondria can serve as platforms for pro-inflammatory signaling, though not all pathways have yet been shown to function in neurons and/or glia. PINK1/Parkin activity may act both to initiate pro-inflammatory signaling and to turn off this signaling by mediating the engulfment of damaged mitochondria by autophagosomes (78). Furthermore, activation of NF-κB signaling by PINK1/Parkin can curtail inflammation (94, 112–114) and upregulate mitophagy (116). Collectively, these studies suggest that pathways for mitophagy and inflammatory signaling interact in a delicate balance that can self-regulate under basal conditions. However, in response to stress, this balance may be destabilized and actively contribute to disease progression.

## Mitophagy in PD

PD is a progressive neurodegenerative disorder, affecting more than 10 million people worldwide. The disease is characterized by loss of dopaminergic neurons in the substantia nigra, triggering motor symptoms such as tremors, rigidity, and bradykinesia. PD is also characterized by nonmotor symptoms, including cognitive impairment, sleep disturbances, and autonomic dysfunction (117).

**Figure 2:**
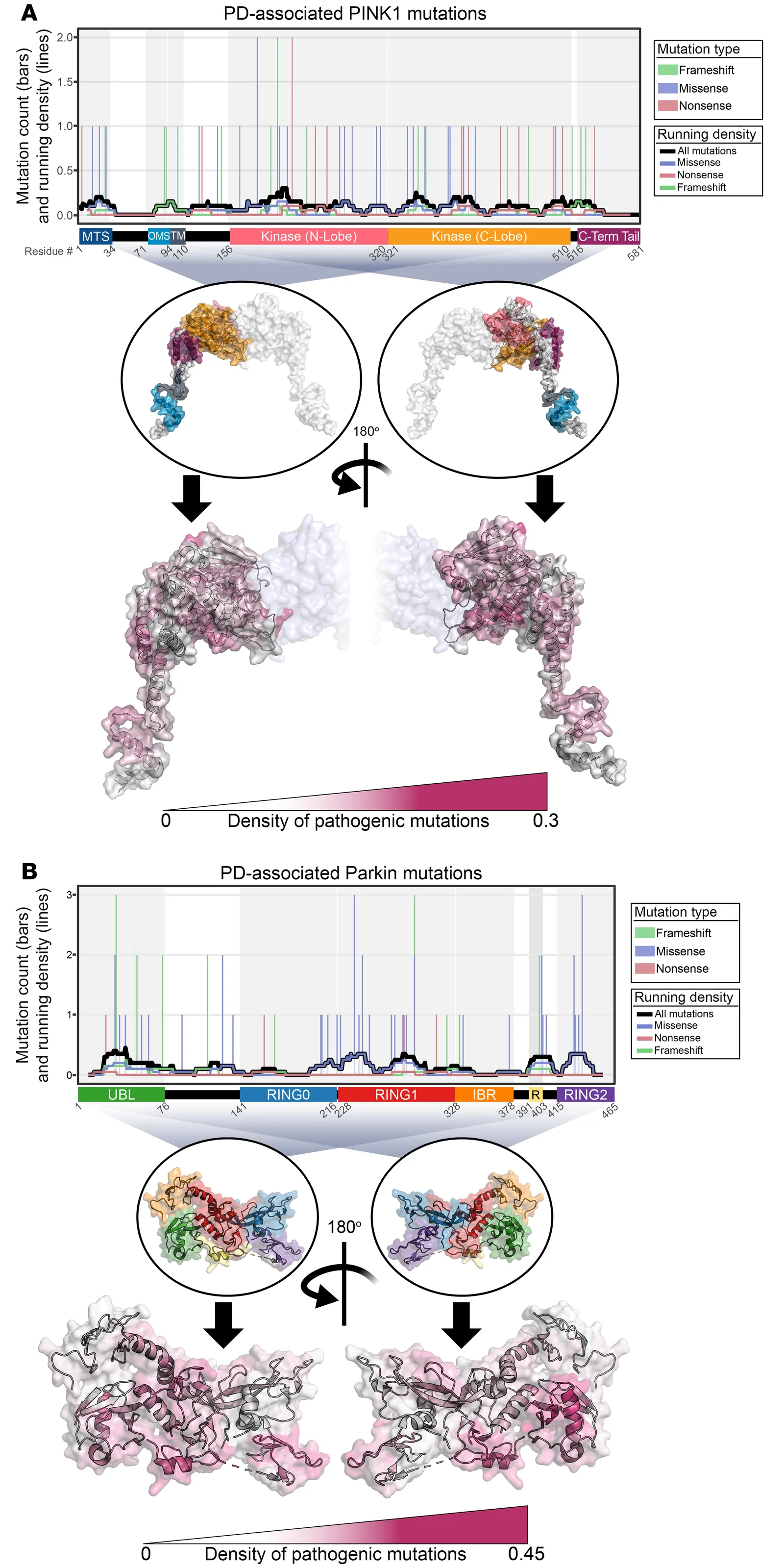
Mutational maps of PINK1 and Parkin. Mutations in PINK1 (**A**) and Parkin (**B**) that are classified in ClinVar (208) as pathogenic, likely pathogenic, or possibly pathogenic are shown. Mutation type refers to the impact on the translated protein; only mutations that change the structure of the protein are included. Bars indicate the number of each kind of mutation at a given amino acid residue; lines indicate running density of each kind of mutation (colored) or total mutation (black) based on a 20-residue window. Domains are colored on the circled structures, as they are on the domain map of each protein, and the heatmap of pathogenic mutations on the protein’s structure represents the normalized overall running density of mutations. PINK1 is illustrated as a dimer, as it is found when stabilized on the mitochondrial outer membrane, with only one PINK1 subunit colorized. PINK1 PDB: 9EIH (126); MTS, mitochondrial targeting sequence; OMS, outer membrane localization signal; TM, transmembrane domain; C-term tail, C-terminal tail. Parkin PDB: 4K95 (134); UBL, ubiquitin-like domain; RING0-2, really interesting new gene domains 0-2; IBR, in-between-ring domain; R, repressor element of Parkin.

Mutations in *PINK1* lead to early-onset PD (EOPD) (Figure 2A) (118–122); patients with such mutations typically respond well to levodopa, a drug that boosts dopamine production (118). In vitro, mutations in *PINK1* impair mitophagy by inhibiting Parkin recruitment and activation at damaged mitochondria (123, 124). Structural studies have helped clarify the mechanistic effects of some of these mutations. A crystallographic study on insect PINK1 mapped approximately 20 disease-causing point mutations that detrimentally affect protein stability or function, altering ATP-binding, substrate-binding, or kinase activity (125). Studies on human PINK1 demonstrated that the N-terminus of the protein is embedded in the barrel of TOMM40 (translocase of the outer mitochondrial membrane 40), while residues 110 through the C-terminus, including the kinase domain, remain cytoplasmic. This cytoplasmic portion of PINK1 homodimerizes and trans-autophosphorylates to open the Ub-binding domain (19, 126). These changes occur in concert with modulation of disulfide bonds that exist both within and between two PINK1 polypeptides to stabilize the proteins internally, as a dimer, and on OMM proteins to which they are anchored (126). These structural insights are beginning to provide insight into why mutations in *PINK1* have distinct clinical presentations; for instance, 10 pathogenic *PINK1* mutations identified across a cohort of patients with PD in Europe and North Africa showed variable age of onset and clinical manifestations (127).

More than 120 mutations causal for autosomal recessive EOPD have been identified in *Parkin*. Most *Parkin* mutations result in disease that is levodopa responsive, again pointing to specific dopaminergic neuron loss (128). However, dementia is not common in patients with *Parkin* mutations, though it is unclear whether this is due to the early-onset nature of Parkin-mediated PD or a true pathological difference between patients with EOPD versus those with sporadic PD (128). EOPD-causing *Parkin* mutations are generally clustered within functional domains of the protein (Figure 2B) but can differentially affect enzyme activity, clinical presentation, or pathology. For example, K161N and K211N mutations both map to the RING0 domain of Parkin, but Parkin^K211N^ is unable to localize to damaged mitochondria while Parkin^K161N^ is recruited, albeit at a lower efficiency than WT Parkin (129). Clinically, most affected patients exhibit the Parkinsonian triad of bradykinesia, rigidity, and resting tremors (130), though age and nature of onset can vary depending on the mutation and possibly on environmental factors (130). Of note, many cases of EOPD caused by a *Parkin* mutation do not display Lewy body pathology, otherwise considered one of the defining features of sporadic PD (131).

Structural studies on Parkin provide insight into how pathogenic mutations differentially affect protein function. Parkin is a member of the RING-between-RING (RBR) family of E3 Ub ligases (132, 133) and contains an additional fourth zinc-binding domain (RING0) that is unique to Parkin and located N-terminal to the canonical RBR core (134–137). There is also an N-terminal Ub-like domain (UBL) (Figure 2B). Early x-ray crystallography studies (134, 135) identified key residues for Parkin function; the RING0 domain blocks the catalytic site in the RING2 domain, the RING1 domain interacts with E2-conjugating enzymes, and the catalytic cysteine responsible for carrying out Parkin’s function as an E3 ligase (Cys431) is in the C-terminal RING2 domain (134). Parkin also harbors an α-helix, termed repressor element of parkin (REP), which is located N-terminal to the RING2 domain (Figure 2B). At baseline, REP acts as a repressor element, rendering Parkin inactive by blocking the site in the RING1 domain at which E2-conjugating enzymes such as UBCH7 interact with Parkin (132, 134). Iterative mutagenesis has shown that Parkin is recruited to Ub before being activated (138). PINK1-dependent phosphorylation of Parkin causes the UBL domain and a short segment of the UBL-RING0 linker (the activator region) to interact with RING0 in a way that displaces RING2, making the catalytic cysteine on RING2 accessible and allowing this domain to carry out ubiquitination of substrates (139).

In an effort to better characterize the in vivo roles of PINK1/Parkin, several groups have generated *Pink1*- or *Parkin*-knockout mice or pigs. Surprisingly, these models exhibit normal mitophagy with minimal loss of dopaminergic neurons (63, 64, 68, 140). One possible reason for this could be compensatory mechanisms for clearance of damaged mitochondria via bulk autophagy or alternative pathways such as BNIP3/NIX–mediated mitophagy (11); alternatively, these observations may reflect species-specific effects (141). Indeed, in nonhuman primate models, knockout of either *PINK1* or *Parkin* induces substantia nigra neurodegeneration with symptoms more analogous to those observed in human patients (67, 68). In rhesus monkeys, cortical neurodegeneration was observed by 1.5 years of age following CRISPR-mediated *PINK1* knockout (67). *Parkin* knockout in the brain of adolescent and older primates led to selective neurodegeneration in the substantia nigra and phospho–α-synuclein pathology (68). Notably, *Parkin^–/–^* primates aged 6–8 years show detectable but less extensive dopaminergic neurodegeneration, whereas the same knockout in primates 22–25 years old results in more extensive neuronal loss (68). These findings suggest an increased reliance on mitophagy with aging. Of note, overexpression of WT PINK1 in primates 22–25 years old increased activated Parkin levels and reduced protein aggregation (68). Together, these studies provide compelling evidence that PINK1/Parkin–dependent mitophagy serves as a protective mechanism against aging-induced mitochondrial damage in primates.

While the roles of PINK1 and Parkin in mitophagy have been studied most extensively, several other PD-associated proteins have also been implicated in mitochondrial quality control. Hyperactive mutations in the kinase LRRK2 that represent one of the most common causes of familial PD have been reported to disrupt mitophagy flux in vivo in mouse models (59) and in vitro in induced pluripotent stem cell–derived (iPSC-derived) human dopaminergic neurons (142). These deficits in mitophagy have been partly attributed to impaired degradation of the OMM adaptor protein, MIRO, induced by pathogenic LRRK2 (143). α-Synuclein, whose aggregation into Lewy bodies and neurites represents the classic neuropathological hallmark of PD, block mitochondrial transport and function (144). Aggregated α-synuclein can promote cardiolipin externalization to the OMM (145), which under normal conditions triggers mitophagy via LC3 recruitment; however, α-synuclein may also compete with LC3 for cardiolipin binding, potentially impairing mitochondrial clearance (146). Heterozygous mutations n the gene encoding β-glucocerebrosidase impact lysosomal function and have been associated with increased mitochondrial stress and reduced mitophagy both in vitro and in postmortem brain samples from patients with PD (147). These studies highlight the broader role of mitophagy in mitochondrial quality control in the context of PD.

## Mitophagy in ALS

ALS is a debilitating neurological disorder primarily impacting motor neurons in the brain and spinal cord. The first clinical symptom is often focal muscle weakness, followed by muscular degeneration and cramps, fasciculations, and bradykinetic movements associated with increased muscle stiffness (148). Approximately 5%–10% of ALS is familial; multiple environmental factors have been implicated as causative for sporadic ALS but conclusive data are lacking (149). *SOD1* was the first causative gene to be identified (150). Since then, mutations in over 40 genes have been associated with ALS (151); among these genes, *OPTN*, *TBK1*, *VCP/p97*, and *SQSTM1* play a critical role in autophagic pathways such as mitophagy.

**Figure 3:**
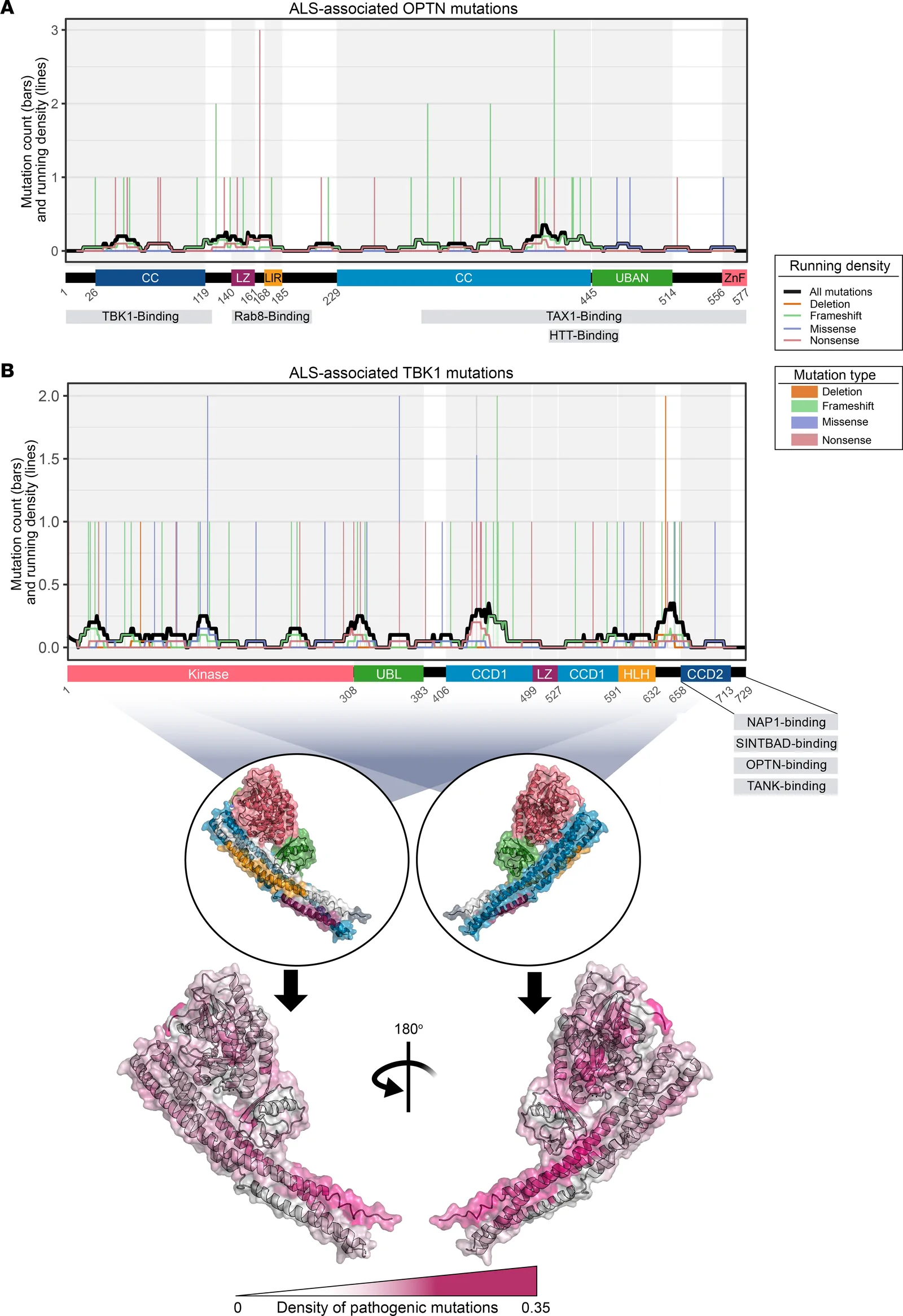
Mutational maps of OPTN and TBK1. Mutations in OPTN (**A**) and TBK1 (**B**) that are classified in ClinVar (208) as pathogenic, likely pathogenic, or possibly pathogenic are shown. Mutation type refers to the impact on the translated protein, and only mutations that change the protein’s structure are included. Bars indicate raw number of each kind of mutation at a given amino acid residue; lines indicate running density of each kind of mutation (colored) or total mutation (black) based on a 20-residue window. Binding sites for pertinent proteins are indicated below the domain maps in gray. OPTN: CC, coiled-coil; LZ, leucine zipper; LIR, LC3-interacting region; UBAN, ubiquitin binding in ABIN and NEMO; ZnF, zinc finger. TBK1 PDB: 4IWO (209).

Mutations in *OPTN* were first identified as causal for primary open-angle glaucoma and subsequently identified in patients diagnosed with ALS (152) (Figure 3A). These mutations included a deletion of exon 5, a homozygous Q398X nonsense mutation, and a heterozygous E478G missense mutation. Since then, several other *OPTN* mutations have been identified in ALS (153); these mutations contribute to 1%–4% of familial ALS and 0.4% of sporadic cases (154). Importantly, both the Q398X and E478G mutations in OPTN affect its Ub-binding domain, impairing mitophagy (23, 152). WT OPTN is rapidly recruited to damaged mitochondria in response to Parkin-dependent ubiquitination of OMM proteins (23). Both the Q398X and E478G mutations disrupt this recruitment, causing the mutant protein to remain cytosolic and attenuating mitochondrial turnover (27). Consistent with this, expression of OPTN E478G in primary neurons inhibited mitochondrial clearance, resulting in the formation of swollen, dysfunctional mitochondria (29). Expression of the OPTN E478G mutant was also found to induce activation of NF-κB and secretion of pro-inflammatory cytokines such as IL-1β, IL-6, and TNF-α (155). Mice infected with lentivirus expressing OPTN E478G exhibit severe cortical inflammation, neuronal degeneration, and pronounced locomotory defects (155). A patient family diagnosed with the OPTN E478G mutation exhibited progressive motor decline, temporal lobe atrophy, and unusual finger deformities, with TDP-43–positive cytoplasmic inclusions observed in spinal and medullary motor neurons (156). Another patient with the homozygous OPTN Q398X mutation displayed degeneration of cortical and spinal motor neurons, atrophy of the temporal and motor cortex, and TDP-43–positive neuronal and glial cytoplasmic inclusions throughout the central nervous system (157).

The recruitment of OPTN to ubiquitinated mitochondria is facilitated by phosphorylation by the kinase TBK1 (26, 27, 158). Mutations in *TBK1* are found in 1%–3% of patients with familial ALS and less than 1% of patients with sporadic ALS (154). Over 90 mutations have been identified in *TBK1* (Figure 3B); these mutations target the dimerization, autoactivation, and kinase domains of the protein or affect its interaction with OPTN (159, 160). One of the best-characterized TBK1 mutations, E696, is a missense variant that impairs its interaction with OPTN, preventing TBK1 recruitment to damaged mitochondria and impairing mitophagy (26, 161). In vivo studies on homozygous TBK1 E696K mice revealed behavioral deficits and progressive motor defects accompanied by degeneration of motor neurons in the spinal cord, muscle denervation, and axon demyelination in the ventrolateral lumbar spinal cord (162). Motor neurons from these mice exhibited disruption of autophagy and accumulation of enlarged lysosomes and fragmented mitochondria (162). Several other ALS-associated mutations in TBK1 have been studied in mammalian cell lines and primary neurons. These mutations disrupt mitophagic flux by blocking autophosphorylation, dimerization, or kinase function (27, 160). ALS-associated mutations in TBK1 have also been shown to impact autophagic clearance of protein aggregates. For instance, in the SOD1^G93A^ ALS mouse model, introducing TBK1 mutations G217R or R228H, associated with familial and sporadic ALS, respectively, accelerated disease onset and caused rapid progression of early motor deficits (163). Reduced phosphorylation of both p62 and OPTN were also observed, resulting in the buildup of protein aggregates in motor neurons (163).

SQSTM1/p62 is a multifunctional scaffolding protein that functions as an adaptor in diverse autophagic pathways, binding to both ubiquitinated proteins and LC3-positive phagophores. Autosomal dominant mutations in *SQSTM1* have been reported in 2%–3% of ALS cases (164). p62 is recruited to damaged mitochondria (165), promoting aggregation of damaged organelles without affecting clearance (23, 24, 166). There is a lack of evidence linking mutations in *SQSTM1* to defective mitophagy in neurons, but in astrocytes, p62 is actively recruited to damaged mitochondria and may facilitate their clearance (78).

Autosomal dominant mutations in the VCP gene are causal for 1%–2% of familial ALS cases (167). VCP is a homohexameric AAA^+^ ATPase that utilizes ATP to disassemble protein complexes and unfold proteins (168). In addition to regulating mitochondrial fusion (169) and respiration (170), VCP has been reported to be recruited to damaged mitochondria to promote PINK1/Parkin–dependent mitophagy (171). VCP can promote the degradation of MFN1/2 upon ubiquitination of the latter by Parkin, thereby facilitating mitochondrial clearance (172, 173). The pathogenic A232E mutation in VCP impairs mitochondrial clearance, resulting in the formation of mitochondrial aggregates in cellular models (173). Mice harboring the A232E mutation show progressive muscle weakness and bone abnormalities and develop extensive TDP-43 pathology in muscle and brain (174).

Mitophagy pathways operating independently of PINK1/Parkin have also been implicated in neuronal homeostasis in the context of ALS. For instance, in the SOD1^G93A^ ALS mouse model, FUNDC1 levels are lower in the spinal cord, thereby impairing mitophagy and contributing to neuronal apoptosis (175). Overexpression of FUNDC1 rescued these defects and improved motor function and survival (175). Taken together, these findings suggest that deficits in PINK/Parkin–dependent and –independent mechanisms contribute to ALS pathogenesis.

## Mitophagy in AD

AD, the most common form of dementia, is a progressive neurodegenerative disorder characterized by extracellular deposition of Aβ plaques and intracellular aggregates of hyperphosphorylated tau protein, leading to synaptic dysfunction and neuronal loss (176). Evidence of mitochondrial dysfunction was first noted in hippocampal pyramidal neurons in AD patient brains, including increased accumulation of mtDNA and mitochondrial proteins in the cytoplasm (177). Although genes directly involved in mitophagy are not known to be mutated in AD, mitophagy is impaired by mutant tau and Aβ aggregates (72, 178). Elevated levels of tau in the brains of patients with AD correlate with increased accumulation of mitophagy markers, such as TOMM20 and COX IV (178). Neurons derived from iPSCs expressing the familial AD mutant (APP^V717L^) or the sporadic AD apolipoprotein E4 (APOE4)/E4 variant exhibit increased mitochondrial fragmentation and a reduction in mitophagic flux coupled to reduced levels of the mitophagy-associated proteins TBK1, AMBRA1, FUNDC1, and MUL1 (72). Importantly, in *App/Ps1* mouse models of AD, induction of mitophagy by urolithin A improved mitochondrial health and aided in reduction of Aβ plaques and cognitive impairment (72). Similarly, overexpression of PINK1 in the mAPP mouse model of AD (179) or Parkin overexpression in sporadic AD patient-derived fibroblasts (180) was able to restore mitophagy. Collectively, this work suggests that boosting mitophagy may have a positive therapeutic effect on patients with AD.

## Therapeutic approaches to restore mitophagy

Efforts to increase mitophagic flux have been reported to promote neuronal health and survival across multiple models. These observations have stimulated development of targeted interventions aimed at enhancing mitochondrial quality control.

USP30 is a deubiquitinating enzyme (DUB) that represses mitophagy by deubiquitinating OMM proteins. USP30 localizes to the OMM and deubiquitinates several substrates of Parkin, including MIRO1, TOMM20, and MUL1, thus inhibiting mitophagy (181). Loss of USP30 in *Drosophila* or mouse models of PD rescues mitophagy as well as motor defects and dopamine loss from the brain (182, 183). These observations motivated small molecule discovery efforts to target USP30. Currently the best-characterized chemical inhibitor of USP30 is MTX115325, developed by Mission Therapeutics. This drug, now in phase I clinical trials, is central nervous system penetrant and highly selective, with an IC_50_ of 12 nM (183). In an α-synuclein mouse model of PD, oral administration of the drug for 10 weeks prevented the degeneration of dopaminergic neurons, rescuing loss of dopamine and its metabolites HVA and DOPAC. In vitro, MTX115325 increases mitophagy in a dose-dependent fashion, indicating that boosting mitophagy may be a key mechanism to restore neuronal loss in PD. Several other drugs targeting USP30 are undergoing preclinical studies, including MF-094 and MF-095, developed by Mitobridge (184), and CMPD-39, developed by the University of Sheffield (185).

Pharmacological strategies aimed at boosting PINK1 activation or Parkin function have emerged as promising approaches to enhance mitochondrial quality control in neurodegenerative disease. MTK458 is a small molecule developed by Mitokinin that activates PINK1 by stabilizing the PINK1-TOM complex, thereby boosting mitophagy (186). Administration of this brain-penetrant compound to an α-synuclein mouse model of PD promoted clearance of α-synuclein aggregates and rescued the associated locomotor and behavioral deficits (186). AbbVie, which has now acquired Mitokinin, has initiated a phase I clinical trial for the PINK1 activator under the name ABBV-1088 (NCT06414798). PR-364 from Progenra is a selective small molecule activator of Parkin shown to boost mitochondrial biogenesis and mitophagy in cardiomyocytes, protecting against mortality after myocardial infarction (187). While this compound has not been studied in the context of neurodegeneration, the mechanism is relevant and holds promise for preclinical studies. A series of Parkin activators have been developed by Biogen that promote the activity of the E3 ligase in cell-free biochemical experiments but failed to promote Parkin translocation in cells and had little effect on mitophagy (188).

Naturally occurring compounds have also been reported to boost mitophagy. Urolithin A is a small compound present in pomegranates, nuts, and berries that has been shown to stimulate mitophagy in muscle, increasing muscle function in mice (189). Intraperitoneal administration of urolithin A in aged mice diminished neuroinflammatory responses and age-associated neurological deterioration; these improvements were linked to increased PINK1/Parkin mitophagy and improved mitochondrial biogenesis and function (94). Urolithin A has also been shown to induce p62-mediated lysophagy in the mouse retina, thereby restoring proteostasis and neuronal survivability (190). A recent study showed that isoginkgetin, a bioflavonoid derived from *Ginkgo biloba*, can induce mitophagy by stabilizing the PINK1-TOM complex on the OMM. In iPSC-derived motor neurons from patients with ALS, isoginkgetin boosted mitophagy, reduced neurite swelling, and increased neuronal survival (191). Other naturally occurring compounds shown to boost mitophagy include actinonin, an antibiotic derived from *Streptomyces* spp. (192) and the NAD^+^ precursor nicotinamide mononucleotide (193).

## Challenges remaining and the way ahead

Both preclinical and clinical studies aimed at enhancing mitophagy specifically, or autophagy pathways more broadly, offer promise for development of therapeutic approaches going forward. However, the low levels of autophagy observed in neurons make the pharmacological upregulation of this process particularly challenging (194–196). Low levels of autophagic flux in neurons are due, at least in part, to higher expression of negative regulators of autophagy such as MTMR5/2, Rubicon, and Bassoon. Consistent with this, depletion of Rubicon and MTMR2 was shown to increase mitophagy under basal conditions and during oxidative stress (30). Thus, small molecule development targeting negative regulators of autophagy may offer a fresh approach to upregulating mitophagy in the context of neurodegenerative disease.

Screens to identify modifiers of mitophagy in other contexts, such as cancer (197), may yield additional candidates relevant to treatment of neurodegenerative diseases. However, an important concern is the ability of these compounds to cross the blood-brain barrier. For instance, the mitophagy-inducing agent nilotinib (198) exhibited poor central nervous system penetration, with only ~0.2–0.3% of its serum concentration detected in cerebrospinal fluid, a limitation that likely contributed to its lack of efficacy in clinical trials for PD (195). Brain-targeted delivery platforms such as lipid nanoparticles (199) or antibody/receptor–mediated transport vehicles (200) can overcome this limitation by more effectively delivering compounds across the blood-brain barrier.

One critical challenge that remains is the lack of effective tools to measure mitophagy in humans. Fluorescent reporters such as mitoKeima (201) and mitoQC (202) are useful tools in cellular or animal studies but are inapplicable in human trials. Further, these probes are dependent on intact lysosomal function, meaning if lysosomal function is compromised, as is increasingly appreciated in neurodegenerative diseases (203), then reduced signal cannot be reliably attributed to impaired mitophagic flux alone. Efforts to bridge this gap have focused on biomarkers, such as phospho-Ser65 Ub levels in postmortem brain samples or cerebrospinal fluid (204, 205), as indirect readouts of mitophagic flux. A complementary approach involves measuring mtDNA heteroplasmy, i.e., the ratio of mutant to normal mitochondrial DNA in a cell. Impaired mitophagy allows mutant mtDNA to accumulate, while efficient mitochondrial removal keeps heteroplasmy low (206, 207). While this approach has been studied in model systems, whether heteroplasmy shifts caused by changes in mitophagy are detectable in human biofluids remains a question.

Thus, while multiple challenges remain in targeting mitophagy in neurodegenerative diseases, it is highly encouraging that advances in basic research are beginning to translate into viable approaches for modulating disease progression in devastating neurodegenerative disorders.

## Conflict of interest

The authors have declared that no conflict of interest exists.

## Funding support

This work is the result of NIH funding, in whole or in part, and is subject to the NIH Public Access Policy. Through acceptance of this federal funding, the NIH has been given a right to make the work publicly available in PubMed Central.

Aligning Science Across Parkinson’s (ASAP-000350) through the Michael J. Fox Foundation for Parkinson’s Research to ELFH.
ASAP-028260 to BB.
NIH/NINDS National Research Service Award 1F31NS143320-01 to JFR.

## Version 1 09/01/2026

Electronic publication
